# The cyclic guanosine monophosphate synthase-stimulator of interferon genes pathway as a potential target for tumor immunotherapy

**DOI:** 10.3389/fimmu.2023.1121603

**Published:** 2023-04-21

**Authors:** Rui Chen, Mingxia Liu, Quanhong Jiang, Xiangbo Meng, Junmin Wei

**Affiliations:** ^1^ Department of Medical Oncology, Qilu Hospital, Cheeloo College of Medicine, Shandong University, Jinan, Shandong, China; ^2^ Department of Biochemistry and Molecular Biology, Department of Immunology, School of Basic Medical Science, Tianjin Medical University, Tianjin, China; ^3^ Advanced Medical Research Institute, Meili Lake Translational Research Park, Cheeloo College of Medicine, Shandong University, Jinan, Shandong, China

**Keywords:** cGAS-STING, tumor, immune regulation, immunotherapy, clinical application

## Abstract

Cyclic guanosine monophosphate–adenosine monophosphate (cGAMP) synthase (cGAS) detects infections or tissue damage by binding to microbial or self-DNA in the cytoplasm. Upon binding DNA, cGAS produces cGAMP that binds to and activates the adaptor protein stimulator of interferon genes (STING), which then activates the kinases IKK and TBK1 to induce the secretion of interferons and other cytokines. Recently, a series of studies demonstrated that the cGAS-STING pathway, a vital component of host innate immunity, might play an important role in anticancer immunity, though its mechanism remains to be elucidated. In this review, we highlight the latest understanding of the cGAS-STING pathway in tumor development and the advances in combination therapy of STING agonists and immunotherapy.

## 1 Introduction

Innate immunity, as the first line of defense against pathogenic infections, depends on pattern recognition receptors that identify pathogen-associated molecular patterns (PAMPs) and damage-associated molecular patterns (DAMPs). PAMPs and DAMPs, which include aberrant RNA or DNA, RNA-DNA hybrids, or cyclic dinucleotides from either pathogens or the microbiome, elicit a series of immune responses (1, 2). The immune system serves as an essential defense mechanism against cancer, as it recognizes and eliminates neoplastic cells during immunosurveillance. Therefore, strong innate immunity is critical for adaptive antitumor immunity (3).

Recent evidence suggests that tumor-derived DNA is the primary DAMP driving the host antitumor immune response (4). However, although immune checkpoint inhibitor-based immunotherapy has become the standard therapy for many cancers, its efficacy depends on the antitumor immune effect triggered. The critical cytoplasmic DNA pattern recognition receptor, cyclic guanosine monophosphate–adenosine monophosphate synthase (cGAS), binds to double-stranded DNA (dsDNA) and activates stimulator of interferon (IFN) genes (STING), which in turn induces the release of type I IFNs and other inflammatory cytokines that trigger an adaptive immune response (5–7). Therefore, this pathway has great potential as a target for improving immunotherapeutic efficacy. In this review, we summarize the latest mechanism and roles of the cGAS-STING pathway in tumor development and highlight associated treatments in combination with the latest research.

## 2 cGAS-STING signaling pathway

### 2.1 Activation of cGAS

The catalytic activity of cGAS is activated when it binds to dsDNA, which triggers a conformational change. The C-terminal nucleotidyltransferase of cGAS, which is crucial to its function, consists of a catalytic structural domain, dsDNA recognition domains, and a conserved zinc ion binding site. When the two DNA binding sites are in close proximity, a stable “ladder-like” dimer complex is formed, which activates cGAS (8). The complex then alters the catalytic structural domain to convert GTP and ATP into cyclized 2’3’-cyclic GMP–AMP (cGAMP), which has a higher affinity for STING. While cGAS activation is primarily dependent on dsDNA length, single-stranded DNA, single-stranded RNA, and dsRNA can also bind to cGAS, but they cannot activate the domain (9, 10). The DNA-induced liquid–liquid phase separation mechanism of cGAS is also crucial for its activation. Polyvalent interactions between cGAS and DNA cause the formation of droplets that function as microreactors through which cGAS can be more efficiently activated by DNA. Together with zinc ions, the complex forms liquid droplets, leading to dimerization and activation (11–13). Since liquid–liquid phase separation relies on polyvalent interactions, long DNA activates cGAS more efficiently than short DNA. This also prevents the pseudo-activation of cGAS by limited and short dsDNA, thereby providing an important intrinsic protection mechanism for living cells (14).

In addition, intracellular stresses such as oxidative stress or DNA damage can lead to the release of manganese ions, which are preferred by cGAS for the efficient synthesis of cGAMP (15). Previous studies by Tao Li’s team (16, 17) have shown that the stress granule protein G3BP1 pre-assembles cGAS through liquid–liquid phase separation, enabling a rapid response to DNA stimulation. They also discovered that the G3BP1 inhibitor, epigallocatechin gallate, can prevent cGAS activation. The combination of cGAS and dsDNA triggers the production of cGAMP, a second messenger and agonist of STING, which is considered a critical first step (11).

### 2.2 Sensing of DNA damage by cGAS

Cancer cells are typically characterized by an abundance of cytoplasmic dsDNA derived from various sources, such as extracellular DNA, mitochondrial DNA, and genomic DNA, which is not commonly observed in normal cells.

The induction of non-apoptotic tumor cell death by exogenous stimuli can release unprogrammed extracellular DNA, apoptosis-derived DNA, and exosomes (18). This DNA can be degraded by 3-prime repair exonuclease 1 (TREX1), which prevents aberrant nucleic acid recognition. However, tumor-derived DNA that remains undegraded can activate the cGAS-STING pathway, induce type I IFNs, and drive immune cell recruitment (19). Moreover, mitochondrial endonuclease G deficiency, mitochondrial protease YME1L deficiency, or chemotherapy drug induction can cause mitochondrial DNA stress, leading to resistance to TREX1 clearance through the reactive oxygen species pathway and mitochondrial DNA accumulation (20–22). The accumulated DNA can enter the cytoplasmic matrix through macropores in the outer mitochondrial membrane *via* BAX/BAK and voltage-dependent anion channel proteins VDAC1 and VDAC3, activating the cGAS-STING pathway (18, 23–25). Furthermore, tumor cells with genomic instability are prone to chromosome loss or division during mitosis due to oncogenic mutations, oxidative stress, and hypermetabolism. This can cause DNA leakage in the form of micronuclei, chromatin fragmentation, and free telomeric DNA, which can also be sensed by cGAS and affect antitumor immunity (26, 27).

### 2.3 cGAS-mediated signaling cascade

cGAMP is a second messenger that binds to STING, which is anchored on the endoplasmic reticulum (ER). cGAMP binding induces a conformational change in STING, which subsequently translocates from the ER to the Golgi apparatus. This process is thought to liberate the STING carboxyl terminus to subsequently recruit and activate TANK-binding kinase 1 (TBK1) and IFN regulatory factor 3 (IRF3) *via* a phosphorylation-dependent mechanism. STING also activates NF-κB, which functions together with IRF3 to activate the transcription of type I IFNs and other cytokines. TBK1, a kinase that binds to the C-terminal tail of STING *via* a conserved PLPLRT/SD motif, tightly controls the activation of IRF3. Upon binding, TBK1 phosphorylates Ser366 within the C-terminal tail, resulting in the upregulation of IRF3. IRF3 is then phosphorylated and dimerized by TBK1, before entering the nucleus to activate the transcription of type I IFNs and IFN-stimulated genes (ISGs) (28). IFNs have been shown to have multiple effects on different immune cells, and recent evidence suggests that the effectiveness of antitumor immunotherapy largely depends on IFN signaling (29).

**Figure 1:**
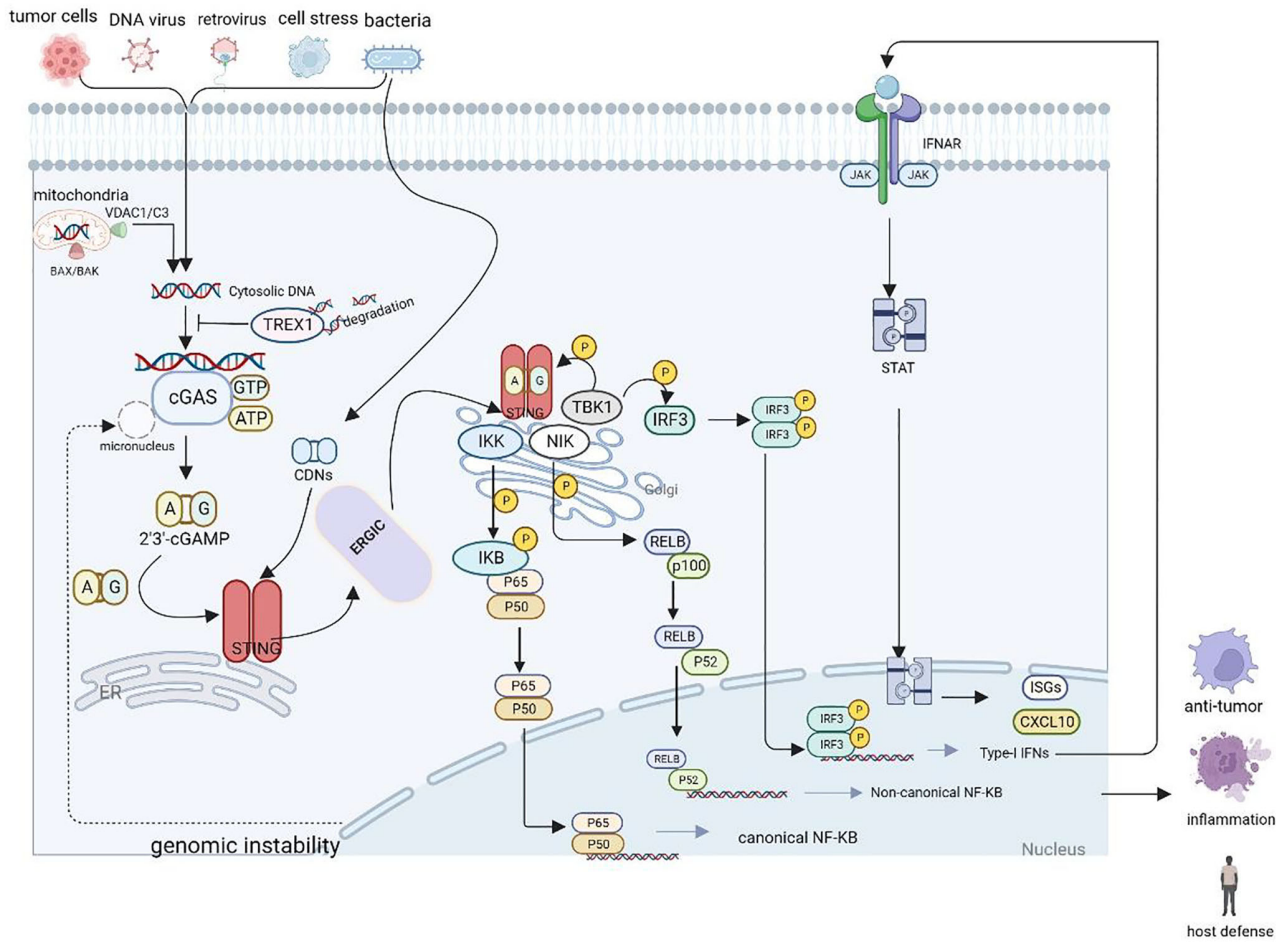
Schematic representation of the cGAS-STING cascade. DNA derived from various sources including viruses, dying tumor cells or nuclei and mitochondria binds to and activates the cytosolic DNA sensor cGAS. cGAS catalyzes the synthesis of 2′3′-cGAMP in the presence of ATP and GTP, and then 2′3′-cGAMP binds to the ER adaptor STING, which also can be activated by CDNs derived from bacteria. Upon activation, STING translocate from the ER to the Golgi compartments, where it activates TBK1 and IKK, which phosphorylate IRF3 and IκBα respectively.

The STING pathway also activates NF-κB-dependent transcription. The IκB kinase phosphorylates IκBα, an inhibitor of NF-κB, which leads to its degradation *via* the ubiquitin–proteasome pathway, thereby freeing NF-κB to enter the nucleus and trigger canonical NF-κB signaling. NF-κB collaborates with IRF3 and other transcription factors to release IFNs (e.g., IFNα and IFNβ) and inflammatory cytokines (e.g., tumor necrosis factor [TNF], interleukin [IL]-1β, and IL-6). Moreover, mitogen-activated protein kinase 14 phosphorylates the NFκB2/p100 subunit that complexes with RelB, a proto-oncogene that is part of the NF-κB subunits (12). Phosphorylated p100 undergoes proteasomal degradation to form p52, which forms a heterodimer with RelB, triggering non-canonical NF-κB signaling (12, 30, 31). Notably, cGAS-STING also promotes non-canonical NF-κB by inducing p52-RelB nuclear translocation. Thus, the cGAS-STING pathway acts as a key negative regulator of the STING effector mechanism by suppressing STING-driven signaling of type I IFNs and canonical NF-κB signaling. As a result, the cGAS-STING signaling pathway may have important implications in limiting cancer immune escape and metastasis (32). **Figure 1** illustrates the cGAS-STING signaling cascade.

### 2.4 cGAS-STING and autophagy

Research suggests that when STING is activated on the ER–Golgi intermediate compartment, it binds to microtubule-associated protein 1-light chain 3 on the autophagic membrane, inducing lipidation and STING degradation. This ultimately terminates signal transduction and triggers autophagy, which removes DNA and viruses from the cytoplasmic matrix *via* a mechanism independent of TBK1 or IFN (33). This implies that autophagy induction by STING may be a primordial function of the cGAS pathway. Importantly, autophagy may prevent sustained STING phosphorylation, but not NF-κB activation (34). This process also contributes to the destruction and removal of microbial DNA present in the cytoplasm *via* enzymatic degradation within lysosomes (35, 36). Additionally, cGAS-STING activation mediated by the cGAS-STING-TBK1-IRF3 assembly complex can occur directly in the autophagosome (34). Clinical trials that have tested autophagy inhibitors in combination with other chemotherapeutic agents (37) indicate that autophagy inhibitors combined with pathway agonists have great potential in antitumor immunotherapy.

### 2.5 cGAS-STING and senescence

The state in which defective cells enter permanent cell cycle arrest, known as cellular senescence, has been historically regarded as a tumor-suppressive mechanism. This process is largely attributed to the accumulation of damaged DNA and activation of the p53 or p16 pathway (38). Senescent cells secrete inflammatory cytokines, growth factors, and proteases; this is known as the senescence-associated secretory phenotype (SASP) and can inhibit tumorigenesis (39).

There is evidence that suggests the cGAS-STING pathway is initiated by self-derived cytoplasmic DNA substrates produced by genotoxic stress-induced cellular senescence, leading to STING-mediated production of SASP in an NF-κB-dependent manner (40, 41). This indicates the potential of the STING pathway in regulating senescence (42). Short-term exposure to SASP factors drives the recruitment of immune cells to clear pre-malignant cells and senescent cells, thereby preventing tumorigenesis (42, 43). Therefore, modulating the activity of the cGAS-STING pathway could benefit cancer immunotherapy and inhibit cancer progression by promoting antitumor immunity and inducing SASP.

## 3 The cGAS-STING signaling pathway and antitumor immunity

The cGAS-STING pathway plays a significant role in regulating tumorigenesis. Its activation in tumor cells increases their immunogenicity by inducing IFNα and IFNβ, which could bind to IFN receptors on immune cells in an autocrine or paracrine manner. IFNα can activate the JAK-STAT pathway, promoting the expression of ISGs such as IFIT1, ISG15, and CXCL10. Among these, CXCL10 is crucial in recruiting CD8+ T cells and enhancing cytotoxicity. On the other hand, IFNβ can stimulate the mobilization of immune cells such as dendritic cells (DCs) and T cells, and induce the presentation of tumor-associated antigens or neoantigens by MHC molecules on the surface of DCs to CD8+ T cells, thus eliciting an antitumor immune response (27, 44).

Pattern recognition receptors are present on immune cells such as DCs, macrophages, T cells, and natural killer (NK) cells in the tumor microenvironment. Damaged nucleic acids released from dying tumor cells, regarded as DAMPs, can activate the cGAS-STING pathway and induce the release of type I IFNs, enhancing antitumor immunity (45, 46). DCs are one of the most important antigen-presenting cells that produce type I IFNs *via* the activated cGAS-STING pathway in their cytoplasm. These IFNs bind to IFN receptors on the surfaces of their own or neighboring DCs, as well as on other immune cells, in a paracrine or autocrine manner. This induces the expression of MHC-I and co-stimulatory molecules, promotes the maturation of DCs, cross-presentation of tumor antigens, and enhances the response of antitumor cytotoxic T lymphocytes (CTLs). Thus, type I IFNs produced by DCs exhibit a combination of innate and adaptive immunity (47, 48). Additionally, these IFNs promote the production of chemokines such as CXCL9, CXCL10, and CXCL11, which enhance the homing in of antigen-presenting cells, movement, and migration of CD8+ T cells and NK cells (47). The STING-dependent expression of type I IFNs induces an established antitumor immune response, with STING-IRF3 signaling required for the initiation of tumor-specific CD8+ T cell responses. Tumors with reduced DNA repair capacity and high mutational burden often present as immunologically “hot” tumors and exhibit better immunotherapeutic outcomes. Recent studies have shown that STING activation leads to antitumor effects independent of the type of immune cells but dependent on activation of the NF-κB pathway. CD8+ T cell depletion in melanoma B16-bearing mice did not affect intra-tumor injection of cGAMP’s ability to reduce tumor load, suggesting that STING activation promotes antitumor activities independent of CD8+ T cells. STING-dependent type I IFN signaling has also been reported to induce antitumor NK cell activation (45, 49). While normal somatic cells undergo senescence as their telomeres shorten with each cell division, 90% of tumor cells are immortalized due to continuously extending telomeres. 6-thio-dG, a nucleoside analogue, is detected by telomerase and incorporated into nascent telomeres, which become imbalanced and cause massive DNA damage in tumor cells. These DNA fragments are captured by DCs, which activate type I IFN to further promote the proliferation and activation of antigen-specific T cells and enhance antitumor immunity (50). Epigenetic modulation is also vital in tumor immune escape; Wu et al. demonstrated that inhibition of the histone demethylase KDM5B activates type I IFN through the cGAS-STING pathway to suppress the anti-immune response (51). Activation of DNA immune recognition is also accompanied by AKT pathway upregulation. The HER2-AKT oncogenic pathway inhibits cGAS-STING pathway-mediated DNA immune recognition by suppressing STING-TBK1 interaction, as well as TBK1 K63-type ubiquitination, thereby inhibiting type I IFN production, cellular senescence, and apoptosis in tumor cells (52).

The expression of cGAS and STING has been observed to be significantly reduced in CD8+ T cells derived from cancer patients. Furthermore, in mouse models, T cell therapy was less effective when CD8+ T cells lacked cGAS or STING. Endogenous activation of cGAS-STING was found to enhance the antitumor immune response by promoting the differentiation of stem cell-like CD8+ T cells, which offers novel approaches to improving the efficacy of chimeric antigen receptor T-cell therapy (53).

## 4 Tumor suppression of the cGAS-STING pathway

Tumor cells have developed intrinsic inhibitory mechanisms to prevent activation of the cGAS-STING axis, which enables them to evade immune surveillance.

### 4.1 Epigenetic mechanisms

The expression of cGAS and STING is often epigenetically altered in many tumors. For instance, in lung cancer, the expression of cGAS and STING is repressed by the epigenetic regulator DNMT1. Knockdown of the long non-coding RNA NEAT1 reduces the enrichment of DNMT1 at the promoters of cGAS and STING, which in turn promotes the production of IFNβ, CXCL10, and CCL5 (54). Hence, it is essential to unravel epigenetic mechanisms that impede the innate immune response and identify potential targets to enhance the antitumor immune response.

### 4.2 Aberrant post-translational modifications and degradation

Zhang et al. reported that the deubiquitinase USP35 directly interacts with STING (55). Knockdown of USP35 led to enhanced phosphorylation of STING, TBK1, and IRF3, improved endogenous interactions between STING and TBK1, and promoted the expression of type I IFNs. High levels of USP35 were associated with reduced CD8+ T cell infiltration and poor prognosis in patients with ovarian cancer. In addition, the β-galactoside-binding protein Gal-9 was found to be associated with shorter survival in patients with nasopharyngeal carcinoma. The carbohydrate recognition domain 1 of Gal-9 directly interacts with the STING C-terminus and recruits TRIM29, which mediates the K48 chain of STING ubiquitination, leading to STING degradation (56). Therefore, it is crucial to develop cGAS-STING agonists with individualized dosing regimens to enhance antitumor immunity.

## 5 The cGAS-STING pathway and tumor biotherapy

**Figure 2:**
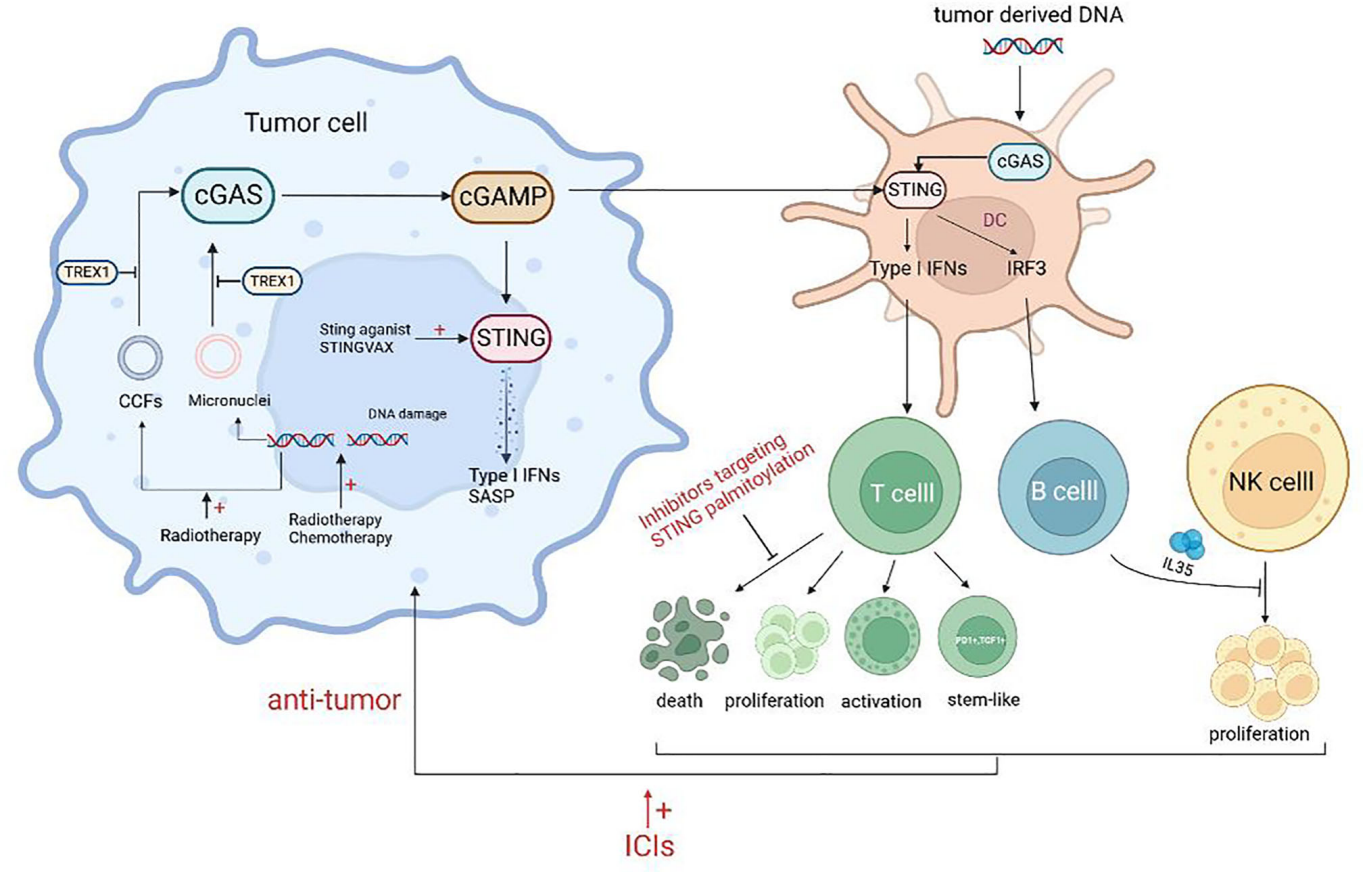
Schematic representation of the agonists of cGAS-STING and antitumor immunity. Treatment such as radiotherapy and chemotherapy can lead to DNA damage, resulting in increased free intracellular DNA and damaged chromatin, and lead to the formation of micronuclei which can activate the cGAS-STING pathway. Released inflammatory mediators act on T cells to promote the proliferation, activation, and stem-like changes of T cells. However, some STING agonists can promote the death of CTLs and the secretion of NK inhibitory factors by B cells, which is not conducive to antitumor immunity. Combining ICIs or targeting adverse effects will improve the efficacy of STING agonists.

The potential for cGAS-STING to trigger innate immunity within the tumor microenvironment makes it a promising target for tumor therapy (57, 58). **Figure 2** illustrates the relationship between cGAS-STING agonists and antitumor immunity. Ongoing preclinical and clinical studies have demonstrated that various therapeutic modalities can synergize with cGAS-STING agonists to remodel the tumor immune microenvironment and enhance antitumor effects (47).

### 5.1 STING agonists

Over the past decade, numerous human STING agonists have been developed, some of which, including cyclic dinucleotides (CDNs) and their derivatives, as well as non-CDN-based STING agonists and their analogues, have entered clinical trials (listed in **Table 1**) (100). Notably, several experiments have demonstrated that intra-tumor administration of cGAMP and other CDN stimuli results in significant antitumor responses in animal models such as 4T1 breast cancer, squamous cell carcinoma, CT26 colon cancer, and B16F10 melanoma. These responses were accompanied by increased secretion of TNFα and various chemokines, providing strong evidence for the involvement of the cGAS-STING pathway in antitumor immunity (101). However, the hydrophilicity of STING agonists may result in reduced antitumor efficacy, making effective drug delivery systems such as nanocarriers, particles, hydrogels, and others essential (12). Recently, Li et al. reported that nanoparticles of the novel polymer agonist PC7A, coupled with cGAMP, specifically target STING within immune cells and show great therapeutic potential in a tumor-bearing mouse model (102).

**Table 1:** STING agonists that have been included in clinical studies or preclinical studies.

Drugs	Mechanisms	Disease	Test phase	Clinical trial registry number and Reference
Hafnium oxide nanoparticles	STING1 agonists	solid tumors	Phase III	*NCT02805894* *NCT04892173* *NCT04505267* *NCT04862455* *NCT04484909* (59)
IMSA-101	STING1 agonists	solid tumors	Phase II	*NCT04020185*
MK-1454	STING1 agonists	squamous cell carcinoma of the head and neck; solid tumors; lymphomas	Phase II	*NCT04220866* *NCT03010176* (60)
exoSTING	STING1 agonists	solid tumors; meningeal carcinomatosis; glioblastoma	Phase I/II	*NCT04592484* (61)
ADU-S100	STING1 agonists	solid tumors; lymphomas	Phase I	*NCT03937141* *NCT02675439* *NCT03172936 (* 62)
BI 1387446	STING1 agonists	solid tumors	Phase I	*NCT04147234 (* 63)
BMS-986301	STING1 agonists	solid tumors	Phase I	*NCT03956680*
DN-015089	STING1 agonists	solid tumors	Phase I	*CTR20212462*
E-7766	STING1 agonists	Solid tumors; lymphomas; bladder tumor	Phase I	*NCT04144140* *NCT04109092 (* 64)
GSK-3745417	STING1 agonists	solid tumors	Phase I	*NCT05424380* *NCT03843359*
HG-381	STING1 agonists	solid tumors	Phase I	*NCT04998422*
KL-340399	STING1 agonists	solid tumors	Phase I	*NCT05387928* *NCT05549804*
MK-2118	STING1 agonists	solid tumors; lymphomas	Phase I	*NCT03249792*
ONO-7914	STING1 agonists	solid tumors	Phase I	*JPRN-jRCT2031* *210530*
SB-11285	STING1 agonists	squamous cell carcinoma of the head and neck; solid tumors; melanoma	Phase I	*NCT04096638*
SNX-281	STING1 agonists	solid tumors; lymphomas	Phase I	*NCT04609579*
SYNB-1891	STING1 agonists	solid tumors; lymphomas; metastatic tumors; inflammatory bowel disease	Phase I	*NCT04167137* (65)
TAK-500	STING1 agonists	solid tumors	Phase I	*NCT05070247 (* 66)
TAK-676	STING1 agonists	squamous cell carcinoma of the head and neck; solid tumors; non-small cell lung cancer; triple negative breast cancer	Phase I	*NCT04879849* *NCT04420884* *NCT04541108 (* 67–69)
XMT-2056	STING1 agonists	colorectal cancer; HER2 mutant non-small cell lung cancer; HER2-positive breast cancer; HER2-positive gastric cancer; solid tumors	Phase I	*NCT05514717 (* 70, 71)
AdVCA0848	STING1 agonists	tumors	Preclinical studies (72)	
ALG-031048	STING1 agonists	colorectal cancer; hepatocellular carcinoma; melanoma	Preclinical studies (73)	
anti-STING antibody	STING1 Inhibitors	tumors	Preclinical studies (74)	
C-176	STING1 agonists	tumors	Preclinical studies (75)	
GF3-002	STING1 agonists	tumors	Preclinical studies (76)	
CF501/RBD-Fc	STING1 agonists	tumors	Preclinical studies (77)	
CS-1010	STING1 agonists	tumors	Preclinical studies (78)	
CS-1018	STING1 agonists	tumors	Preclinical studies (78)	
CS-1020	STING1 agonists	tumors	Preclinical studies (78)	
diABZI-2	STING1 agonists	tumors	Preclinical studies (79)	
GSK-532	STING1 agonists	tumors	Preclinical studies (80)	
H-151	STING1 agonists	tumors	Preclinical studies (81)	
IACS-8779	STING1 agonists	tumors	Preclinical studies (82, 83)	
IMGS-501	STING1 agonists	solid tumors	Preclinical studies (84)	
MSA-1	STING1 agonists	tumors	Preclinical studies (85)	
MSA-2	STING1 agonists	tumors	Preclinical studies (85)	
ONM-501	STING1 agonists	tumors	Preclinical studies (86)	
PC7A	STING1 agonists	tumors	Preclinical studies (87)	
SB-02024	VPS34 inhibitor	tumors	Preclinical studies (88)	
SHR1032	STING1 agonists	tumors	Preclinical studies (89)	
SN-011	STING1 agonists	tumors	Preclinical studies (90)	
SOMCL-18-202	STING1 agonists	tumors	Preclinical studies (91, 92)	
SP23	STING1 agonists	tumors	Preclinical studies (93)	
SR-717	STING1 agonists	tumors	Preclinical studies (94)	
SR-8314	STING1 agonists	tumors	Preclinical studies (95)	
SR-8541A	STING1 agonists ENPP1 inhibitors	tumors	Preclinical studies (96)	
STING1 agonists ADC	STING1 agonists	tumors	Preclinical studies (97)	
STING1-TLR9	STING1 Inhibitors; TLR9 agonists	tumors	Preclinical studies (98)	
IACS-8803	STING1 agonists	tumors	Preclinical studies (99)	

There is increasing evidence of the involvement of non-CDN drugs in cGAS-STING activation. For instance, DMXAA, a flavonoid STING agonist with anti-angiogenic properties, has been shown to induce the expression of IFNβ and enhance the proliferation and infiltration of CD8+ T cells (58). However, despite its promising preclinical data, DMXAA failed to demonstrate efficacy in a phase III clinical trial for non-small cell lung cancer (103). Subsequently, it was discovered that although DMXAA activates the STING pathway in mice, it does not have the same effect in humans or rats (104).

In response to this phenomenon, a study explained that the protective immune effect provided by S100, a STING agonist, was mainly driven by CD8+ T cells. However, the response to the tumor showed a bell-shaped curve with the increase of S100 concentration, indicating the importance of dose control. High concentrations of S100 led to T cell death, which may be one of the reasons for the lack of clinical therapeutic effect of S100 (105). Similarly, Wu et al. showed that STING agonists could induce T cell death in an IFN-independent way and that inhibitors targeting STING palmitoylation effectively blocked STING-mediated T cell death *in vitro*. However, these inhibitors also suppressed other IFN-dependent STING pathway activations, such as macrophages, thus resulting in a new requirement for STING inhibitors for precise targeting (106) (**Figure 2**).

Despite the unsatisfactory clinical results of DMXAA, these valuable works have prompted efforts to design analogues with a higher affinity for hSTING (11). Quan et al. have shown that α-mangostin, a derivative of DMXAA, can activate hSTING more effectively than mSTING (107). These findings suggest that the rational design of DMXAA analogues will stimulate the emergence of novel antitumor therapies.

### 5.2 STING agonists combined with other treatments

The aforementioned findings suggest that activation of the STING axis alone may not be sufficient for immunotherapeutic tumor clearance. Several studies have demonstrated that the STING agonist cGAMP induces the expression of IL-35 in B cells through an IFN-independent and IRF3-dependent mechanism. This results in the inhibition of NK cell proliferation and attenuation of their antitumor response. To enhance the efficacy of STING agonists, the combination of these agents with IL-35 antagonists may be a viable approach (106) (**Figure 2**).

The field of tumor immunotherapy has made significant progress through the introduction of immune checkpoint inhibitors (ICIs). A study by Sivick et al. demonstrated that the combination of PD-1 and CTLA4 antibodies increased the sensitivity of mouse tumors to ICI treatment, leading to significant tumor control (105). However, the lack of T-cell infiltration makes most tumors insensitive to ICI treatment. STING agonists can induce the expression of ISGs (such as CXCL9 and CXCL10) and the infiltration of CTLs, which can convert “cold tumors” into “hot tumors” and overcome resistance to ICIs (46, 108). Additionally, the STING axis can enhance the sensitivity of tumor cells to NK cells and CTLs (109). STING agonists also inhibit the depletion of MHC molecules on tumor cells, which is vital for tumors to evade immune surveillance (47). Therefore, the combination of STING agonists and ICIs has the potential to enhance the sensitivity of tumor cells to the latter (**Figure 2**). Solid tumors with DNA mismatch repair deletion or microsatellite high instability (dMMR/MSI-H) produce a substantial amount of tumor antigens that enable PD-1 antibodies to enhance the antitumor response (110). It has been found that dMMR/MSI-H tumors can activate the cGAS-STING pathway by regulating the activity of the nucleic acid Exo1, and that immunotherapy is ineffective in similar tumors with abnormal cGAS-STING activity. Thus, the expression levels of cGAS-STING-encoding genes can also be used to predict dMMR tumor response to immunotherapy (111, 112).

STING agonists are important in cancer vaccine adjuvants, as they are used to enhance tumor-specific immunity and overcome tolerance (47, 108). The innate immune response activates antigen-presenting cells, promoting the immunogenicity of tumor-associated antigens (47). STINGVAX, the first STING-based cancer vaccine designed using granulocyte–macrophage colony-stimulating factor (GM-CSF)-secreting cancer cells and CDNs, has been found to induce a superior antitumor response *in vivo* compared to unformulated GM-CSF-secreting tumor cell vaccines in models of CT26 colon cancer, upper gastrointestinal squamous cell carcinoma (SCCFVII), and pancreatic cancer (PANC02) (113).

## 6 Therapeutic modalities targeting the cGAS-STING pathway

### 6.1 Targeting DNA damage

Radiotherapy, chemotherapy, and other targeted therapies are frequently utilized in clinical cancer treatments due to their ability to activate the cGAS-STING pathway by inducing DNA damage, ultimately promoting an antitumor immune response.

In addition to directly destroying DNA strands and causing tumor cell death, radiation therapy can activate the cGAS-STING pathway through various mechanisms. These mechanisms include DNA damage-induced micronucleus formation, cytoplasmic chromatin fragmentation induced by aging, and the ZBP1-MLKL pathway-dependent release of mitochondrial DNA (11, 114, 115). It is worth noting that activation of the cGAS-STING pathway through radiation therapy is dependent on the radiation dose. Low doses of radiation can prevent TREX1 activation, enabling cGAS-STING induction. In contrast, high doses of radiation (20-30 Gy) often inhibit pathway activation by increasing TREX1 (116) (see **Figure 2** for a summary of the mechanisms of cGAS-STING activation by radiation therapy).

In clinical practice, drugs targeting DNA damage repair are also utilized. The DNA damage response (DDR) kinases, ATM and ATR, work together during DNA replication and repair to initiate DNA damage sensing and activate the DDR pathway. The ATM inhibitor, KU-60019, induces microglial cytosolic DNA accumulation and promotes the secretion of proinflammatory cytokines in a STING-dependent manner (117). Combining ATM inhibitors with PD-L1 antibodies has been shown to increase therapeutic efficacy in mice bearing ARID1A-deficient tumors (118). Poly ADP-ribose polymerase 1 (PARP1), an enzyme that interacts with damaged DNA, is crucial for mediating the DDR pathway, regulating chromatin structure, and maintaining genomic stability. PARP inhibitors are approved for the standard treatment of breast or ovarian cancers with BRCA mutations. Research has demonstrated that PARP inhibitors effectively activate the STING pathway, inducing CD8+ T cell aggregation in patients with BRCA1/2-mutated triple-negative breast cancer (119). Furthermore, in combination with the SHP2 agonist lovastatin and ATR inhibitors, PARP1 can activate the cGAS-STING pathway and play an antitumor role in colon cancer and PBRM1-deficient renal clear cell carcinoma (120, 121).

Checkpoint kinase 1 (CHK1) functions as a DNA damage monitor during replication, detecting any abnormalities and regulating replication-driven activation while also acting on S-phase progression. Prexasertib, a CHK1 inhibitor, has been found to activate the STING pathway in small cell lung cancer cells, including in immunocompetent models. This activation results in increased chemokine levels, promoting the activation of CTLs (122, 123).

### 6.2 Targeting DNA replication

Some drugs that target DNA replication damage can activate the cGAS-STING pathway and induce antitumor immunity. Topotecan, Teniposide, and etoposide, which are topoisomerase inhibitors, can cause replication fork collision and abnormal DNA activation, leading to the activation of STING signaling and its downstream NF-κB and type I IFN pathways in a cGAS-dependent or independent manner, thereby promoting the infiltration of DCs and CD8+ T lymphocytes and inhibiting tumor growth (35, 123–125). Antimetabolites such as hydroxyurea upregulate chemokine expression in a cGAS-STING-dependent way by inducing DNA damage, inhibiting ribonucleoside diphosphate reductase, and blocking replication forks (126). In addition, cross-linking agents such as cisplatin can form covalent adducts on cellular DNA, which alter DNA structure and hinder replication forks, inducing DNA damage and activating the cGAS-STING pathway (127).

### 6.3 Spindle assembly

The microtubule-targeting agent paclitaxel has recently been found to interfere with chromosome segregation and induce type I IFNs and TNFα, promoting the production of pro-apoptotic vesicles *via* the cGAS-STING pathway in primary human breast tumors (128). This mechanism enhances antitumor immunity.

## 7 Conclusion

Immunotherapies for cancer, represented by PD-1/PD-L1 antibodies and CAR-T cell therapy, have been widely used in clinical practice, but many patients still do not benefit from these treatments. PD-1/PD-L1 antibodies and CAR-T cell therapy mainly intervene in the adaptive immune process of cancer patients; however, therapies targeting innate immunity are still lacking. As a vital component of host innate immunity, cGAS-STING and its downstream cytokines, especially type I IFNs, link innate and adaptive immunity, making STING an attractive target for cancer immunotherapy. A growing preclinical study has demonstrated that the cGAS-STING pathway plays pivotal roles in DC-mediated antigen cross-presentation and subsequent priming of tumor-specific CD8+ T cells. Although numerous human STING agonists have been developed and entered clinical trials, the efficacy of STING agonist monotherapy is poor, highlighting the need to conduct more in-depth research on the mechanism of the cGAS-STING pathway in the tumor environment. Recent studies have found that the cGAS-STING pathway and its agonists may suppress the function of antitumor immune cells, including CD8+ T cells and NK cells (105, 106). Therefore, further research is required to explore the role of the cGAS-STING pathway in different tumor-infiltrating immune cells in specific tumors. In terms of drug design, designing STING agonists with higher affinity and tumor-specific carriers can also help improve their clinical effectiveness. With the advancement of basic and clinical research, targeting the cGAS-STING pathway has the potential to provide new strategies and drug combinations for cancer immunotherapy.

## Author contributions

RC and MXL wrote the manuscript, QHJ collected and analyzed data, XBM and JMW conceived and designed this review. All authors contributed to the article and approved the submitted version.
